# GPC3 and PEG10 peptides associated with placental gp96 elicit specific *T* cell immunity against hepatocellular carcinoma

**DOI:** 10.1007/s00262-023-03569-2

**Published:** 2023-11-06

**Authors:** Lijuan Qin, Jiuru Wang, Fang Cheng, Jiamin Cheng, Han Zhang, Huaguo Zheng, Yongai Liu, Zhentao Liang, Baifeng Wang, Changfei Li, Haoyu Wang, Ying Ju, Huaqin Tian, Songdong Meng

**Affiliations:** 1 CAS Key Laboratory of Pathogen Microbiology and Immunology, Institute of Microbiology, Chinese Academy of Sciences (CAS), Beijing, China; 2 University of Chinese Academy of Sciences, Beijing, China; 3 Foshan Hospital of TCM, Guangdong, China; 4 Senior Department of Hepatology, The Fifth Medical Center of PLA General Hospital, Beijing, China

**Keywords:** Cytotoxic *T*-cell, Epitopes, GPC3, Hepatocellular carcinoma, PEG10, Placental gp96

## Abstract

The placenta and tumors can exhibit a shared expression profile of proto-oncogenes. The basis of placenta-derived heat shock protein gp96, which induces prophylactic and therapeutic *T* cell responses against cancer including hepatocellular carcinoma (HCC), remains unknown. Here, we identified the associated long peptides from human placental gp96 using matrix-assisted laser desorption/ionization-time-of-flight and mass spectrometry and analyzed the achieved proteins through disease enrichment analysis. We found that placental gp96 binds to numerous peptides derived from 73 proteins that could be enriched in multiple cancer types. Epitope-harboring peptides from glypican 3 (GPC3) and paternally expressed gene 10 (PEG10) were the major antigens mediating anti-HCC *T* cell immunity. Molecular docking analysis showed that the GPC3- and PEG10-derived peptides, mainly obtained from the cytotrophoblast layer of the mature placenta, bind to the lumenal channel and client-bound domain of the gp96 dimer. Immunization with bone marrow-derived dendritic cells pulsed with recombinant gp96-GPC3 or recombinant gp96-PEG10 peptide complex induced specific *T* cell responses, and *T* cell transfusion led to pronounced growth inhibition of HCC tumors in nude mice. We demonstrated that the chaperone gp96 can capture antigenic peptides as an efficient approach for defining tumor rejection oncoantigens in the placenta and provide a basis for developing GPC3 and PEG10 peptide-based vaccines against HCC. This study provides insight into the underlying mechanism of the antitumor response mediated by embryonic antigens from fetal tissues, and this will incite more studies to identify potential tumor rejection antigens from placenta.

**Supplementary Information:**

The online version contains supplementary material available at 10.1007/s00262-023-03569-2.

## Introduction

Aberrant expression, mutations, and somatic alterations in certain oncogenes and/or tumor suppressor genes have been detected in various human malignancies [1]. The placenta is a heterogeneous and invasive tumor-like organ that invades the uterus to enable the exchange of gases, nutrients, hormones, and other molecules between the maternal and fetal blood as well as provides a barrier that protects the fetus from maternal immune attack [2, 3]. Placenta-specific trophoblast cells exhibit various similarities with cancer cells such as a rapid proliferation ability, epithelial–mesenchymal transition and invasion, cell–cell fusion, induction of angiogenesis, and immune escape [4]. In addition, the mechanisms regulating the functions of trophoblastic and malignant cells are similar. Ferretti et al. [5] reported that activation of the phosphatidylinositol phosphoinositide 3-kinase/AKT axis is a central feature of signaling pathways to achieve proliferative, migratory, and invasive processes in trophoblasts and cancer cells. A recent study reported that human placental tissues contain extensive somatic mutations; the mutation rate per trophoblast cluster is similar to that in childhood cancers, which are primarily induced by mutations in the uterus [6]. Furthermore, the decidual microenvironment that regulates trophoblasts shows many similarities with the tumor microenvironment, such as supporting tumor cell survival including immune cells, oxygen levels, and energy metabolism [7].

The heat shock protein (HSP) gp96, the most abundant chaperone in the endoplasmic reticulum (ER), can modulate innate and adaptive antitumor immune responses. As an essential master chaperone, gp96 is involved in trafficking of Toll-like receptors and alpha integrins to regulate innate immunity as well as *T*- and *B*-cell development [8, 9]. In terms of antigen-specific immunity, tumor-derived gp96-peptide complexes or cell-based gp96–Ig-secreting vaccines elicited specific *T* cell immunity against parent tumors in both rodent models and clinical trials [10, 11]. Gp96 has been proposed to exert multiple roles in *T*-cell activation, with its unique ability to bind a variety of tumor-associated antigens (TAAs) and disease-associated antigens (DAAs) peptides for antigen presentation to major histocompatibility complex (MHC) molecules playing a key role in this process [12–15]. We previously identified a gp96-associated peptide in human liver tumor tissues; this peptide homolog was found to bind to human leukocyte antigen (HLA)-A11 molecules [16, 17]. In addition, we and others showed that cellular gp96 interacts with MHC-I and transporter associated with antigen processing, and gp96 and calreticulin in the ER constitute a relay line for transferring associated peptides to MHC-I molecules for *T*-cell recognition [18, 19]. These studies demonstrated the ability of gp96 to capture and delivery antigens to MHC molecules to generate antigen-specific *T*-cell responses.

Because of the similarity in antigen expression between cancer and embryonic tissues, gp96 extracted from the placenta may bind carcinoembryonic antigens and/or proto-oncogene antigens. We previously showed that placenta-derived gp96 (Pgp96) can serve as a prophylactic and therapeutic vaccine against several cancers, including transplantable melanoma and breast tumors in mice [20]. Moreover, placenta-derived peptides bound to gp96-pulsed dendritic cells (DCs) and generated specific CD8^+^ *T* cell responses against melanoma and Lewis lung cancer in mouse models [21]. A clinical trial was recently initiated to test the safety and therapeutic efficiency of placental gp96-peptide complexes against non-small cell lung cancer and hepatocellular carcinoma (HCC) (No. ChiCTR2100052023). In this study, we isolated gp96-associated peptides from the human placenta, identified the *T* cell epitopes through peptidome analysis using mass spectrometry (MS), and explored the mechanism of placental antigen-mediated immunotherapeutic effects against HCC. These results may facilitate the design of vaccines and *T* cell immunotherapies against cancer.

## Materials and methods

### Mice

HLA-A2.1/Kb (HLA-A2) transgenic mice were maintained in the laboratory [22]. BALB/c-nu mice were purchased from SPF (Beijing, China). All animals were maintained in a specific pathogen-free animal facility and studied at 6–10 weeks of age. Animal experiments were approved by the Research Ethics Committee of the Institute of Microbiology, Chinese Academy of Sciences (approval number PZIMCAS2011001). Except for BMDCs induction, female HLA-A2 mice were used in all immunization experiments.

### Cell culture

HepG2 and *T*2 cell lines were kindly provided by Prof. X Ye (IMCAS, Beijing, China) and Prof. B Gao (IMCAS, Beijing, China), respectively. SK-Hep-1, PANC-1, A549 were obtained from ATCC (Manassas, VA, USA). PANC-1 cell line were cultured in Dulbecco’s modified Eagle’s medium (HyClone, Logan, UT, USA) supplemented with 10% fetal bovine serum (FBS). SK-Hep-1, A549 and HepG2 cell lines were cultured in high-glucose Dulbecco’s modified Eagle’s medium supplemented with 10% FBS. *T*2 cell line was cultured in Roswell Park Memorial Institute 1640 (Gibco BRL, Paisley, UK)) with 10% FBS. All culture media contained 100 U/mL penicillin and 100 μg/mL streptomycin. The HepG2 and SK-Hep-1 cell lines mainly used were authenticated by STR profiling.

### Preparation of placental gp96 and recombinant gp96 proteins

Placental gp96 proteins (Pgp96) of human were extracted as previously described [20]. Placental tissues were obtained from two healthy placentas of normal delivery. Briefly, after grinding the placental tissue, the supernatant was collected and subjected to ammonium sulfate precipitation to obtain the sediment. The dissolved precipitate was purified using ConA-Sepharose affinity chromatography, followed by anion exchange chromatography. Endotoxin levels were determined using the Limulus Amebocyte Lysate assay (< 1 EU/mg) (BioWhittaker, Walkersville, MD, USA).

Soluble recombinant human gp96 (Rgp96) was isolated as previously described [23]. Briefly, recombinant human heat shock protein gp96 constructs were subcloned into the pFastBac1 vector and expressed using the Bac-to-Bac Baculovirus expression system. After filtration and concentration, the supernatant was collected and purified using a HiTrap Q column (GE Healthcare, Little Chalfont, UK). After desalination and concentration, the purity of proteins was greater than 95%, as determined using sodium dodecyl sulfate–polyacrylamide gel electrophoresis (SDS-PAGE).

### Detection and identification of peptides bound to human placental gp96 by mass spectrometry

Bulk polypeptide fraction was isolated from placental gp96 samples by acid treatment release method [17]. Prior to mass spectrometry (MS) analysis, polypeptide samples were freeze-dried with a centrifuge concentrator, redissolved in 100 μL water, sonicated for 5 min, and centrifuged at 14000 × *g* to obtain the supernatant. The supernatant was enriched with a polypeptide chip (L121 mesoporous silicon wafer) and detected using matrix-assisted laser desorption/ionization (MALDI). To identify the peptide sequences, the eluents of all samples were combined, lyophilized, and redissolved in 15 μL of 0.1% formic acid, and the peptide sequences were identified using a nanoLC-Q EXACTIVE (Thermo Fisher Scientific). Analytical separation was performed using gradients of H_2_O/formic acid 100%/0.1% (solvent *A*) and CH_3_CN/formic acid 100%/0.1% (solvent *B*). The gradient was run as follows: 0 min 4% *B*, then to 8% *B* at 8 min, 22% *B* at 58 min, 32% *B* at 70 min, 90% *B* at 71 min, 90% *B* at 78 min at a flow rate of 300 nL/min. The tandem MS scan range was 300–1,600 m/z, and dynamic exclusion time was 40 s.

The peptide mixture was analyzed using liquid chromatography-tandem mass spectrometry (LC–MS/MS). The SEQUEST HT search engine of Thermo Proteome Discoverer (1.4.0.288) was used to search for and identify proteins in the Uniprot-proteome-human (update-20160226) database. The search parameters were as follows: no enzyme was required. The initial maximum allowed mass deviation of the precursor ion was set to 10 ppm, and the maximum fragment ion mass deviation was set to 20 mDa. Methionine oxidation was used as a variable modification. The filter parameter of the results was that the percolator filtered the spectrogram. The delta Cn was < 0.1, and the false discovery rate was set to 1%.

### Peptide synthesis and epitope prediction

The 8–10-mer epitope was predicted using long peptides from two websites: SYFPEITHI (http://www.syfpeithi.de/) and Immune Epitope Database Analysis Resource (http://tools.iedb.org/main/tcell/). Peptides with scores > 20 (for SYFPEITHI) or percentile rank < 0.5 typically have high MHC affinity. All peptides were synthesized by GenScript (Nanjing, China) and were more than 95% pure.

In addition, 5 HLA-DRB1 haplotypes (15:01, 07:01, 08:03, 09:01, 12:02) with > 12% existing frequencies were chosen for HLA-class II epitope prediction. The 12–18-mer epitopes originated from long peptides were calculated with the website (http://tools.iedb.org/mhcii/). Peptides with percentile rank < 1 were identified as strong binders or potential CD4^+^ *T* cell epitopes.

### Preparation and immunization strategy of pulsed-BMDCs vaccines

Mouse bone marrow-derived DCs (BMDCs) were obtained as previously described [24, 25]. Briefly, bone marrow cells were harvested from the femur and tibia of 5–7-week-old mice, and red blood cells were depleted using red blood lysis buffer. The bone marrow cell suspensions were cultured in complete RPMI medium containing 10% heat-inactivated FBS, 20 ng/mL granulocyte macrophage colony-stimulating factor (GM-CSF), 10 ng/mL interleukin-4 (IL-4), and 50 μM 2-mercaptoethanol (Sigma-Aldrich, St. Louis, MO, USA). All cytokines were purchased from PeproTech (Rocky Hill, NJ, USA). After 7 days of culture, nonadherent BMDCs were generated and collected and then, randomly divided into the following different groups according to the experimental design: Pgp96 group: BMDCs incubated with 100 μg/mL placental gp96; Rgp96 group: BMDCs incubated with 100 μg/mL recombinant gp96; GPC3_152–160_ group: BMDCs treated with 100 μg/mL recombinant gp96 and 10 μg/mL GPC3_152–160_; PEG10_229–237_ group: BMDCs treated with 100 μg/mL recombinant gp96 and 10 μg/mL PEG10_229–237_; epitope group: BMDCs treated with 100 μg/mL recombinant gp96 plus 5 μg/mL GPC3_152–160_ and 5 μg/mL PEG10_229–237_; positive control (PC) group: BMDCs loaded with 50 μg/mL tumor cell lysate; DC group: BMDCs alone. To prepare gp96–peptide complexes, gp96 and peptide were mixed and incubated for 10 min at 50 °C, followed by incubation for 30 min at room temperature. All BMDCs were then cultured in the presence of 1 μg/mL lipopolysaccharide (LPS) for 24 h at 37 °C. After incubation, nonadherent mature BMDCs were collected and washed three times with PBS. BMDCs were administered subcutaneously into HLA-A2 female mice on days 0 and 7 (5 × 10^5^ cells in 100 μL PBS/mouse). On day 5 of last immunization, the mice were euthanized and isolated the splenocytes for analyzing by tetramer staining, ELISPOT assay and In vitro tumor killing assay. The same protocol was used in the tumor inhibition experiments with polypeptide-induced *T* cells.

### Animal tumor model and adoptive therapy

BALB/c nude mice, approximately 4–6 weeks of age, were subcutaneously inoculated with 2 × 10^6^ HepG2 tumor cells in 100 μL of PBS in the right flank. Tumor growth was monitored with calipers every other day, and the tumor volume was calculated using the following formula: volume = length × width^2^ × 0.5. At four days after tumor cell inoculation, the tumor-bearing mice were randomly divided into five groups and immunized intravenously in the tail with 3–4 × 10^6^ *T* cells isolated from the spleens of Pgp96 group mice, Rgp96 group mice, epitope or peptide group mice, and PC group mice four times at 3- or 4-day intervals. Adoptive *T* cells were enriched using a CD8^+^ *T* cell Isolation Kit (Miltenyi Biotec, Gladbach Bergisch, Germany). After treatment, the mice were euthanized if the tumor depth exceeded 15 mm.

### Enzyme-linked immunosorbent spot (ELISPOT) analysis

The IFN-γ ELISPOT assays were performed followed by the manufacturer’s instructions (Mabtech, Mariemont, OH). Briefly, isolated mouse splenocytes were added to the wells of 96-well polyvinylidene difluoride plates (BD-Pharmingen, San Diego, CA) in 4–6 replicates and subjected to different stimuli. Typical groups included cells with no stimulant, cells with antigens (tumor cell lysate or peptides), and cells treated with anti-CD3 (2 μg/mL) and anti-CD28 (1 μg/mL) (PC) and incubated at 37 ℃ for the indicated time periods. Spot-forming cells (SFCs) were counted and analyzed using an Immunospot S5 Versa Analyzer (Cellular Technology Limited, Shaker Heights, OH, USA).

#### In vitro* tumor cells killing assay*

Splenocytes were isolated from immunized mice on day 5 after immunization and then, co-cultured with BMDCs (at a 1:20 ratio) that had been incubated with the same treatments as immunization. Each well also contained 20 IU/mL recombinant murine IL-2 (PeproTech). After 5 days of culture, splenic effector cells were collected as effector cells. The target cells (HepG2 or SK-Hep-1) were labeled with 2.5 μM 5-(6)-carboxy-fluorescein succinimidyl ester (CFSE) dye and washed with PBS. We then seeded CFSE-labeled target cells in 100 μL complete RPMI 1640 medium into 96-well round-bottom plates, and effector cells in 100 μL complete RPMI 1640 medium were added to the CFSE-labeled target cells at varying ratios for 4–6 h at 37 °C. The dead cells were labeled with 7-AAD, and cytotoxicity of splenocytes to tumor cells detected using flow cytometry.

### T2-bing assay

The *T*2 cells were incubated with 1 μM human β2m protein (Sigma-Aldrich) and 50 μM peptide and cultured overnight at 37 ℃ in a 5% CO_2_ incubator. The cells were collected and stained with FITC-labeled HLA-A2 antibody and detected using flow cytometry. The fluorescence index (FI) was calculated as described previously [26]; a fluorescence index ≥ 1 was regarded to indicate a high-affinity candidate epitope.

### Tetramer staining and flow cytometry

A tetramer antibody was prepared according to the instructions of the QuickSwitch™ Quant HLA-A^*^ 02:01 Tetramer Kit APC (MBL, Nagoya, Japan). The specific operations were as follows. First, the lyophilized peptide was dissolved in dimethyl sulfoxide to a final concentration of 10 mg/mL. Second, 50 μL of QuickSwitch™ Tetramer was mixed with 1 μL peptide in a microtube, followed by adding 1 μL peptide exchange factor. These steps were repeated for other peptides. Finally, the mixture was incubated for at least 4 h at room temperature in the dark. After the reaction, the tetramer antibody was stored at 4 °C protected from light until use. Peptide exchange was quantified in a flow cytometric sandwich immunoassay according to the manufacturer’s instructions and analyzed using a Calibur flow cytometer (BD Biosciences, USA) and FlowJo Software (Tree Star Inc.). The exchange efficiency of the peptide was 91.9 ± 1.8%, indicating that the tetramer antibody was prepared successfully.

Splenocytes obtained from immunized mice were stimulated with 2 μg/mL peptide for 7 days at 37 ℃ and 5% CO_2_. The cells were harvested and stained with percp-conjugated anti-mouse CD8, FITC-conjugated anti-mouse CD3, and APC-conjugated peptide tetramer antibody. Data were acquired using a Fortessa flow cytometer (BD Biosciences, USA).

As for HLA-A2 blocking study, HLA-A2 mice were depleted of HLA-A2 molecules prior to immunization with BMDCs vaccines. Mice were treated with BB7.2 antibody (anti-HLA-A2) monoclonal antibody at 100 μg per time intraperitoneally 3 times on days -7, -4, -1 before immunization, respectively. When the mice were immunized with the activated BMDCs vaccine, 100 μg of BB7.2 antibody were simultaneously administrated. On day 5 of last immunization, the splenocytes were collected for analysis by tetramer staining.

### Molecular docking analysis

We used Autodock Vina 1.2.2, a computerized protein–ligand docking software, to assess the binding energy and interaction patterns between candidate epitopes and HLA-A2 protein [27]. The three-dimensional structures of proteins GPC3 (PDB ID: AF-P51654-F1), PEG10 (PDB ID: 7LGA, resolution, 1.9 Å), and HLA-A2 (PDB ID: 3HLA, resolution, 2.6 Å) were downloaded from the PDB (http://www.rcsb.org/). The molecular structures of the GPC3_152–160_ and PEG10_229–237_ epitopes were intercepted from them. We first converted all protein and molecular files to PDBQT format, removed all water molecules, and added polar hydrogen atoms. The grid box was centered to cover the domain of each protein and accommodate free molecular motion. The docking pocket was set as a 30 × 30 × 30 Å square pocket, with a grid distance of 0.05 nm for docking. The molecular docking results were analyzed, and models were visualized using PyMol 2.5 and LigPlot + v.2.2.

AlphaFold2 2.2.4 was used to predict the structure of the GPC3 long peptide and PEG10 long peptide bound to gp96 identified using MS. The amino acid sequence of human gp96 was downloaded from NCBI, and the dimer structure was predicted using the AplhaFold2-multimer 2.2.4. The ranked_0 structure file was selected as the final simulation structure of the gp96-dimer protein by comparing the five result files. ZDOCK version 3.0.2 was used to predict the complex model of the receptor and ligand via rigid docking. Interactions between gp96-dimer with GPC3 long peptide, or with PEG10, long peptides were analyzed using Discovery Studio 2019. The final results were visualized and analyzed using PyMol 2.5 and LigPlot + v.2.2.

We downloaded the gp96 dimer structure in different conformations from the RCSB RBD database to analyze its interaction with GPC3 or PEG10 long peptides under different torsion dimer states. The Gp96-NMC/AMP-PNP-bound closed dimer coordinates were obtained from PDB ID 5ULS, GP96-NMC/AMP-PNP-bound “twisted V” dimer coordinates were obtained from PDB ID 2O1U, and GP96-NMC/ADP-bound “twisted V” dimer coordinates were obtained from PDB ID 2O1V. ZDOCK version 3.0.2 was used to predict the complex model of the receptor and ligand via rigid docking. The top 10 ZDOCK scores were analyzed.

### Quantitative real-time PCR

Total RNA was extracted from HepG2 or SK-Hep-1 cells using TRIzol reagent (Invitrogen, Carlsbad, CA, USA) according to the manufacturer’s instructions. RNA was reverse-transcribed using PrimeScript™ RT Master Mix to generate cDNA (TaKaRa, Shiga, Japan). cDNA was analyzed in real-time PCR using a Rotor-Gene Q (Qiagen, Hilden, Germany). The primers used for amplification were as follows: GPC3, (5′-TGGAGAACGTACTGCTTGGTC-3′ forward, 5′-TCTTCTCAGTTTCAGTGGTGG-3′ reverse); PEG10, (5′-GAGAACAGCGGAGAAGGTCC-3′ forward; 5′-CAAAACCCGCTTATTTCACGC-3′ reverse); GAPDH, (5′-GGAGCGAGATCCCTCCAAAAT-3′ forward; 5′-GGCTGTTGTCATACTTCTCATGG-3′ reverse). These primers were used with TB Green Premix Ex Taq (TaKaRa). The quantitative PCR conditions were used as follows: 95 °C for 30 s, followed by 40 cycles of 95 °C for 5 s and 60 °C for 45 s. Amplification of specific transcripts was confirmed from the melting curve profiles generated at the end of the PCR program. The expression levels of target genes were normalized to that of GAPDH and calculated using the comparative cycle threshold (CT) method (2^−ΔΔCT^).

### Western blot assay

Western blot analysis was performed as previously described [23]. Abs against GPC3 (Bioworld Technology, catalog no. BS7410), PEG10 (Bioworld Technology, catalog no. MB0036) were used.

### Isolation of tumor-infiltrating lymphocytes

To isolate tumor-infiltrating lymphocytes (TILs), the tumor was minced and treated with RPMI 1640 containing 2% FBS with 1 mg/mL of collagenase IV, and 100 U/mL of DNase I for 30 min at 37 °C. Then, the TILs were collected through centrifugation on a discontinuous Percoll gradient (GE Healthcare). Isolated cells were then analyzed by flow cytometry.

### PBMCs isolation and amplification

Blood samples from HLA-A2 positive HCC patients were obtained from the fifth medical center of PLA general hospital (Beijing, China) during routine diagnostic procedures after written informed consent was obtained. The study was approved by the Ethics Committee of the fifth medical center of PLA general hospital (approval number KY-2022-12-78-1). Peripheral blood mononuclear cells (PBMCs) were separated from heparinized venous blood by FICOLL PAQUE PLUS (GE) density gradient centrifugation. On day 0, PBMCs (2 × 10^5^/well) were placed in 96-well round-bottom culture plates in 100 μL AIM-V medium (Thermo Fisher) plus anti-CD28 (0.5 μg/mL), anti-CD49d (0.5 μg/mL), and stimulated with GPC10 and PEG10 epitope (1 μg/mL) or long peptide (5 μg/mL) for 5 h at 37 ℃ in a humidified 5% CO2 incubator. After 5 h, 100 μL of complete culture medium with 40 IU/mL IL-2, 20 ng/ml IL-7, 100 ng/ml IL-15,10%, Human Serum (HS) was added to each well. On the day 4, 6 and 8 of induction and amplification in vitro, half of the culture medium was exchanged using the complete medium with 20 IU/mL IL-2, 10 ng/mL IL-7, 50 ng/mL IL-15, and 5% serum. On the ninth day of cultivation, the cell culture medium was completely replaced with a complete medium supplemented with 5% HS and cultured overnight. Then, the cells were collected and stimulated with the indicated peptides for ELISPOT assay.

### Transcriptome analysis

Total RNA was prepared from HepG2 or SK-Hep-1 cells dissolved in TRIzol reagent according to the manufacturer’s instructions. Transcriptome analysis was performed by Novogene Tianjin (Tianjin, China).

### Common database source

All public databases used in this study have been published and can be obtained from the Gene Expression Omnibus database of NCBI, including the human tissue-related transcriptome database GSE1133; HCC-related transcriptome database GSE101685, GSE105130, and GSE121248; and placental single-cell transcriptome database GSE89497.

### Statistical analysis

All data are expressed as the mean ± SD. Statistical variance between groups was analyzed using unpaired two-tailed *t* test and one-way ANOVA analysis used Graphpad Prism 8 software (GraphPad Software, USA). The variance between Kaplan–Meier curves was compared using log-rank test. Differences were considered statistically significant at *P* < 0.05, *P < 0.05, and **P < 0.01.

## Results

### Identification of placental gp96-bound peptides and cytotoxicity testing

**Fig. 1:**
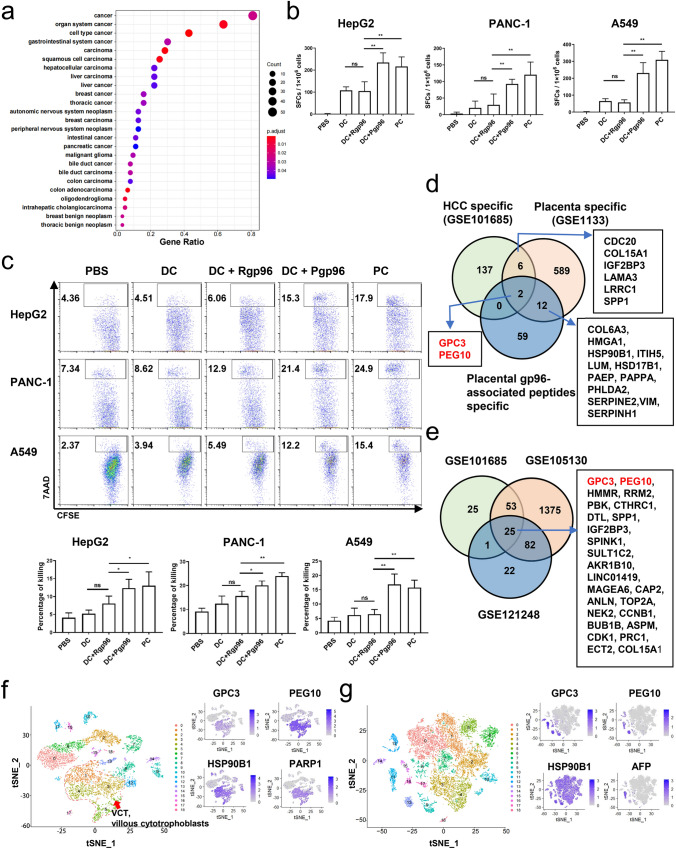
Immunopeptidome analysis of mass spectrometry-identified peptides from human placental gp96. **a** Disease enrichment analysis of proteins from placental gp96-bound peptides identified using mass spectrometry. **b**, **c** Female HLA-A2.1/Kb transgenic mice were immunized with BMDCs pulsed with Pgp96, recombinant gp96 (Rgp96), or tumor lysate as a positive control (PC), or with unpulsed BMDCs (DC) or PBS alone as a negative control. Splenocytes from immunized mice were stimulated with HepG2, PANC-1, or A549 whole-cell lysates, and the number of tumor-specific IFN-γ^+^CD8^+^ *T* cells was evaluated using IFN-γ ELISPOT (**b**). Splenocytes from immunized mice were stimulated and analyzed for cytotoxic activity with CFSE-labeled HepG2, PANC-1, or A549 cells as target cells (**c**). All experiments were conducted three times. The results are presented as the mean ± SD of three mice/group. Ns, not significant; *P < 0.05, **P < 0.01. **d** Venn diagram showing the distribution of shared genes of proteins from placental gp96-bound peptides and genes in the gene expression omnibus public transcriptome dataset for hepatocellular carcinoma (HCC) (GSE101675) and placenta (GSE1133). **e** Venn diagram showing common genes of high-expressed genes in three different datasets for HCC. **f**, **g** *t*-Stochastic neighborhood embedding dimensionality reduction and gene expression of single-cell sequencing data of normal delivery placenta (GSE173193 database) (**f**) and HCC [29] (**g**)

Placenta-derived gp96 (Pgp96) binds intracellular antigenic peptides, including those from proto-oncogenes, that may be shared between the placenta and HCC tumors. Therefore, gp96-peptide complexes were isolated from the human placenta. The Pgp96-associated peptides were extracted, freeze-dried, enriched using L121 mesoporous silicon wafers, and subjected to matrix-assisted laser desorption/ionization-time-of-flight (MALDI-TOF) analysis. The sequences of peptides were detected using Q-Exactive mass spectrometry (MS). We obtained 1130 and 1107 peptides with high confidence levels in two individual experiments. The resulting tandem MS data of the common peptides were searched in the Uniprot-proteome-human (update-20160226) database using the SEQUEST HT search engine of Thermo Proteome Discoverer (1.4.0.288). We identified 73 common proteins in both experiments. Disease enrichment analysis using data from the Disease Ontology database demonstrated that these protein genes were enriched in multiple cancer types, including liver, breast, and pancreatic cancers and so on (Fig. 1a), suggesting that the peptide repertoire from placental gp96 covers a large quantity of tumor antigens.

Then, we examined whether human placental gp96-bound peptides induce tumor-specific *T* cell responses. HLA-A2.1/Kb transgenic mice immunized with Pgp96-pulsed marrow-derived dendritic cells (BMDCs) exhibited a significant increase in HepG2-specific T cells, an HLA-A2-positive HCC cell line (Fig. 1b) and an enhanced cytotoxic effect compared to that in mice immunized with recombinant gp96-pulsed or unpulsed BMDCs (Fig. 1c). Similar results were observed in the human pancreatic adenocarcinoma cell line PANC-1 and lung adenocarcinoma cell line A549. In contrast, tumor antigen-free recombinant gp96 expressed in a baculovirus system did not induce a tumor specific *T* cell response, indicating that the antitumor *T* cell response induced by Pgp96 was attributed to the associated tumor antigens.

We further dissected the HCC antigens from the Pgp96-bound peptides. Through Venn analysis using the Gene Expression Omnibus public transcriptome dataset of HCC (GSE101675) and placenta (GSE1133), we found that two proteins, GPC3 and PEG10, were present in all three datasets (Fig. 1d). Another 25 common genes were detected in three other different datasets for HCC (GSE101685, GSE105130, and GSE121248) and also included GPC3 and PEG10 (Fig. 1e).

We analyzed the sources of GPC3 and PEG10 in the placenta. We traced the expression of these two genes in single-cell sequencing data of sorted placental cells from first- and second-trimester human placentas [28]. The 1567 cells were divided into 13 groups, with each group containing specific high-expression genes (supplementary Fig. S1a and b), suggesting that the overall cell clustering was reliable. Next, we selected the marker genes of different cells based on the results reported by Tsang et al. [29] and re-annotated the data on the above early placental single cells. As shown in Supplemental Fig. S1c, re-annotated cells in the 13 groups included nine cell types. By searching for cell populations highly expressing GPC3 and PEG10 using the FeaturePlot() function of Seurat, GPC3 was found to be highly expressed in stromal and syncytiotrophoblast cells, whereas PEG10 was highly expressed in stromal and cytotrophoblast cells (supplementary Fig. S1d). The GSE173193 dataset provides 10 × Genomics single-cell transcriptome sequencing data for normally delivered placentas. As shown in Fig. 1f (left panel), the cells were divided into 19 groups after *t*-stochastic neighborhood embedding analysis. Analysis of different placental marker genes showed that COL1A1, a marker gene of stromal cells, was not significantly expressed in normal parturient placentas, whereas the GPC3 and PEG10 genes were highly expressed in villous cytotrophoblast cells annotated by the PARP1 gene (supplementary Figs. S1b–d, S2, and Fig. 1f). The same expression pattern was obtained for GPC3 and PEG10 through annotation of the villus cytotrophoblast layer using HLA-G- and KRT7 + double genes in first- and second-trimester human placentas (supplementary Fig. S1b). These results indicate that GPC3- and PEG10-derived peptides bound to gp96 in the mature placenta were mainly derived from the cytotrophoblast layer (Fig. 1f, right panel). Furthermore, we analyzed single-cell RNA sequencing data from HCC tumor specimens based on a study by Sun et al. [30] and found that AFP, a marker gene of HCC, was co-expressed with GPC3 and PEG10 in the same cell (Fig. 1g). These results indicate that the expression of GPC3 and PEG10 is HCC-specific.

### Analysis of immunogenicity of placental gp96-bound GPC3 and PEG10 peptides

**Fig. 2:**
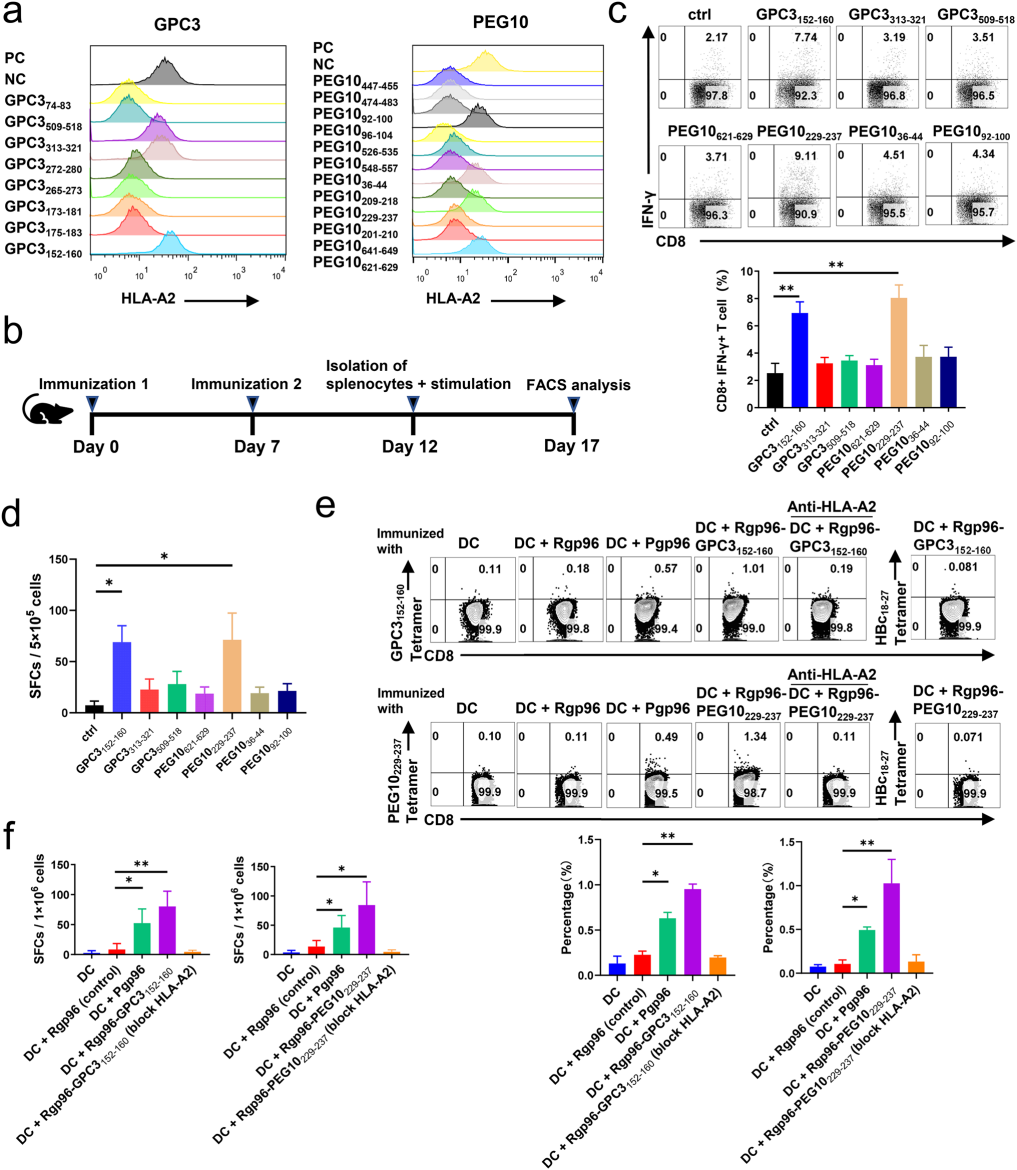
Analysis of HLA-A*0201 restricted epitopes within placental gp96-bound GPC3 or PEG10 peptides. **a** Binding affinity of predicted epitopes was quantified using MHC stabilization assays with T2 cells using flow cytometry. HBc_18–27_ and HBc_82–90_ peptides served as positive (PC) and negative controls (NC). **b** Schema of animal experiments for vaccination strategy. Female HLA-A2.1/Kb mice were subcutaneously immunized with BMDCs pulsed with the mix of three epitopes of GPC3 or four epitopes of PEG10 using Rgp96 as an adjuvant at weeks 1 and 2, respectively. Splenocytes were isolated and stimulated with a single epitope (10 μg/mL) respectively. Epitopes-specific IFN-γ^+^CD8^+^ *T* cells were analyzed using flow cytometry (**c**). The peptide-specific CD8^+^ *T* cells were evaluated by using ex vivo IFN-γ ELISPOT assay (**d**). **e**, **f** Female HLA-A2.1/Kb transgenic mice were subcutaneously immunized with BMDCs pulsed with Rgp96, Pgp96, Rgp96 complexed with GPC3_152–160_ or PEG10_229–237_ epitope, or control BMDCs (DC) at weeks 1 and 2. Isolated splenocytes were stimulated and stained with GPC3_152–160_ or PEG10_229–237_ tetramer and analyzed by flow cytometry. HBc_18–27_ is an HLA-A*0201 restricted epitope of HBV core protein, and HBc_18–27_ tetramer was used as tetramer negative control (NC) to determine the specificity of tetramer staining. The percentage of GPC3_152–160_ or PEG10_229–237_ epitope-specific tetramer^+^CD8^+^ *T* cells in gated total CD8^+^ *T* cells was shown (**e**). Splenocytes were detected in an IFN-γ ELISPOT assay. The number of peptide-reactive cells was represented as spot-forming cells (SFCs) in 1 × 10^6^ splenocytes. The control peptide HBcAg_18–27_ was used for background evaluation (**f**). Data are presented as the mean ± SD of five mice. Ns, not significant; *P < 0.05, **P < 0.01

Four long peptides from GPC3 and six long peptides from PEG10 bound to placental gp96 were found. Bioinformatics analysis revealed that these long peptides contained abundant HLA-A-restricted epitopes (Table 1) and HLA-DR-restricted epitopes (supplementary Table S2), indicating that they may elicit both specific CD8^+^ and CD4^+^ *T* cell responses. Twelve HLA-A*0201 restricted epitopes of PEG10 and eight epitopes of GPC3 were synthesized. As shown in a T2 binding assay (Fig. 2a), three epitopes of GPC3 and four epitopes of PEG10 showed high affinity for HLA-A*0201 molecules. Subsequently, HLA-A2.1/Kb transgenic mice were subcutaneously vaccinated twice with BMDCs pulsed with a mixture of these three peptides of GPC3 or a mixture of the four peptides of PEG10 incorporated with recombinant gp96, and peptide-specific CD8^+^ *T* cells were quantified by analyzing IFN-γ^+^ CD8^+^ *T* cells (Fig. 2b). As shown in Fig. 2c and d, immunized mice showed an activated cytotoxic *T* lymphocyte (CTL) response against epitopes GPC3_152–160_ and PEG10_229–237_ by flow cytometry and enzyme-linked immunosorbent spot (ELISPOT) analysis. The immunogenicity of epitope peptides GPC3_152–160_ and PEG10_229–237_ was confirmed in tetramer (Fig. 2e) and ELISPOT (Fig. 2f) assays. The percentage of GPC3_152–160_ or PEG10_229–237_ epitope-specific tetramer^+^CD8^+^ *T* cells out of total CD8^+^ *T* cells in epitope- or placental gp96-immunized mice was significantly higher than that in recombinant gp96-immunized mice as control. We also blocked the HLA-A2 molecules using BB7.2 antibody, and the GPC3_152–160_ and PEG10_229–237_ specific *T* cells were barely detectable (Fig. 2e and f), indicating that the epitopes were presented to *T* cells by MHC-I molecules. Importantly, mice immunized with Pgp96-pulsed BMDCs also exhibited specific CTL responses to GPC3_152–160_ and PEG10_229–237_, validating that placental gp96 was associated with epitope peptides from GPC3 and PEG10.

**Table 1:** Prediction of HLA-A-restricted epitopes of placental gp96-bound peptides identified by mass spectrometry (Related to Fig. 2)

Gene name	Peptide sequences (position)	HLA type	Epitopes^a^	Position	Score^b^	IEDB percentile rank^d^
GPC3	RDLKVFGNFPKLIMTQVSKSLQVTRIFLQALNLGIEV^d^ (149–185)	HLA-A*02:01	KVFGNFPKL^d^	152–160	21	0.09
			FLQALNLGI	175–183	22	0.21
			RIFLQALNL	173–181	20	1.2
		HLA-A*11:01	KLIMTQVSK	159–167	21	0.19
	NVLLGLFSTIHDSIQYVQKNAGKLTTTIGKLCAHSQQRQYRSAYYPEDLFIDKKVLKV (264–321)	HLA-A*02:01	VLLGLFSTI	265–273	26	0.29
			TIHDSIQYV	272–280	23	0.05
			FIDKKVLKV	313–321	27	0.02
		HLA-A*11:01	STIHDSIQY	271–279	18	0.16
		HLA-A*24:02	AYYPEDLFI	306–314	21	0.06
	HSPLKLLTSMAISVVCFFFLVH (505–526)	HLA-A*02:01	KLLTSMAISV	509–518	25	0.32
	YTNAMFKNNYPSLTPQAFEFVGEFFTDVSL (71–100)	HLA-A*02:01	AMFKNNYPSL	74–83	24	0.77
		HLA-A*24:02	NYPSLTPQAF	79–88	23	0.02
PEG10	AHLATYTEFVPQIPGYQTYPTYAAYPTYPVGFA (620–652)	HLA-A*02:01	HLATYTEFV	621–629	25	0.08
			YAAYPTYPV	641–649	21	1.1
		HLA-A*11:01	QTYPTYAAY	636–644	11	0.19
		HLA-A*24:02	AYPTYPVGF	643–651	23	0.01
			TYTEFVPQI	624–632	24	0.02
			AAYPTYPVGF	642–651	12	0.1
	ALIDQYHEGLSDHIQEELSHLEVAKSLSALIGQCIHIERR^d^ (201–240)	HLA-A*02:01	ALIDQYHEGL	201–210	26	0.16
			ALIGQCIHI^d^	229–237	24	0.16
			GLSDHIQEEL	209–218	24	0.13
	QVQKLTEENTTLREQVEPTPEDEDDDI (33–59)	HLA-A*02:01	KLTEENTTL	36–44	24	0.03
	DHRLVDPHIEMIPGAHSIPSGHVYSLSEPEMAALR (524–558)	HLA-A*02:01	SLSEPEMAAL	548–557	27	0.32
			RLVDPHIEMI	526–535	23	0.32
	NPDMLAPFMAQCQIFMEKSTRDFSVD (89–114)	HLA-A*02:01	FMAQCQIFM	96–104	15	0.56
			MLAPFMAQC	92–100	17	0.26
		HLA-A*11:01	AQCQIFMEK	98–106	15	0.15
		HLA-A*24:02	IFMEKSTRDF	102–111	18	0.16
	VRWLSTHDPNITWSTRSIVFDSEYCRYHCRMYSPIPPSLPP (445–485)	HLA-A*02:01	RMYSPIPPSL	474–483	22	0.15
			WLSTHDPNI	447–455	19	0.55
		HLA-A*11:01	IVFDSEYCR	462–470	21	0.57
		HLA-A*24:02	MYSPIPPSL	475–483	23	0.01
			RMYSPIPPSL	474–483	11	0.05
			TWSTRSIVF	456–464	12	0.12

^a^The position of the epitopes according the Protein Group Accessions (P51654-2 for GPC3, A0A087WXK2 for PEG10). The 8–10-mer epitopes were predicted to bind to HLA-A*02 (A*0201), HLA-A*11 (A*1101), and HLA-A*24 (A*2402) by using different algorithms: the Immune Epitope Database (IEDB) and SYFPEITHI

^b^SYFPEITHI (http://www.syfpeithi.de/bin/MHCServer.dll/EpitopePrediction.htm) score of each peptide. A high score indicates better MHC affinity

^c^NetMHCpan EL 4.1 (http://tools.iedb.org/main/tcell/) score of each peptide. A low percentile rank indicates better MHC affinity

^d^Peptide and epitope were used for anti-HCC animal assay

**Fig. 3:**
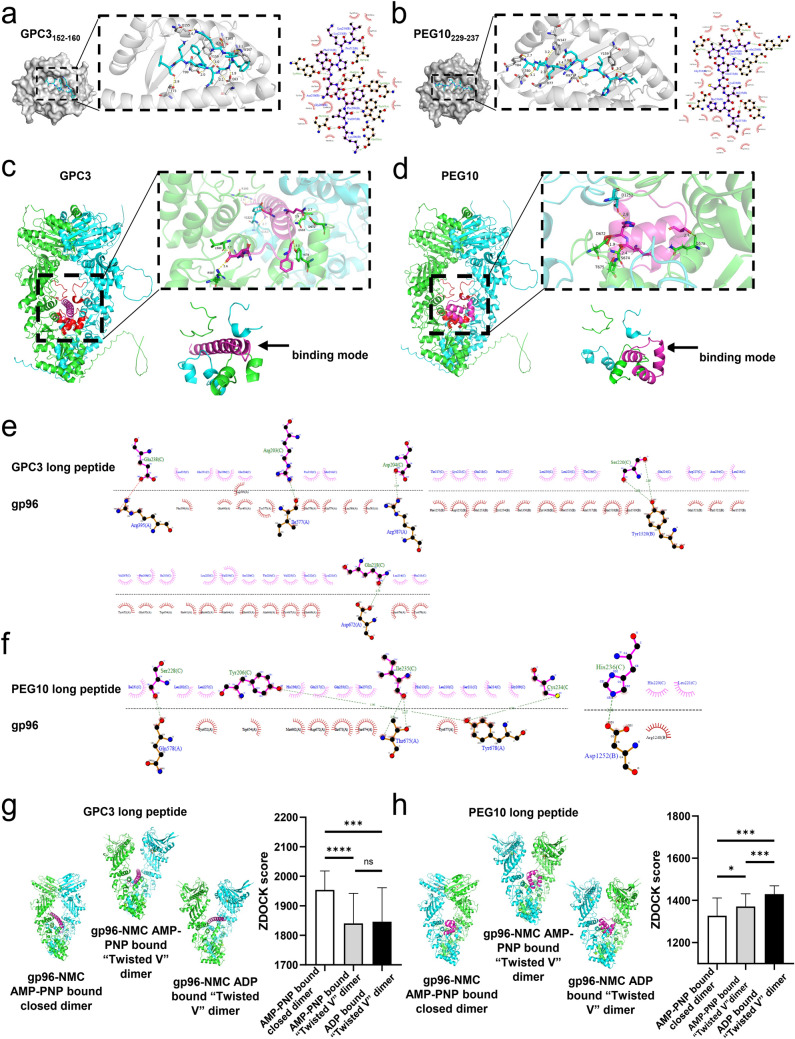
Binding modes of GPC3- and PEG10-derived epitope/HLA-A2 complexes and epitope-harboring long peptide/gp96-dimer complexes. **a**, **b** Models of GPC3_152–160_ epitope/HLA-A2 complexes (**a**) and PEG10_229–237_ epitope/HLA-A2 complexes (**b**) were constructed through molecular docking analysis. The three-dimensional structure and interaction sites of the binding pocket were visualized using PyMol software (left panel), and two-dimensional interactions of epitopes and their targets were exhibited, with hydrogen bonding interactions indicated in green (right panel). **c**, **d** Models of GPC3 long peptide (aa 149–185)/gp96-dimer complexes (**c**) and PEG10 long peptide (aa 201–240)/gp96-dimer complexes (**d**) using ZDOCK. The structures of gp96 and GPC3 and PEG10 long peptide were modeled using AlphaFold2. The left panel shows the three-dimensional structure and interaction sites of the binding pocket. The right panel represents the binding mode of peptides in binding pocket formed by the two loops of the middle-domain and client protein domain of gp96 protein. **e, f** Important binding sites for interaction between gp96-dimer and GPC3 (**e**) or PEG10 (**f**) long peptide analyzed using discovery studio. **g**, **h** Interaction of gp96-dimer with GPC3 (**g**) or PEG10 (**h**) long peptide under different torsion dimer states. The left panel of **g** or **h** shows the interaction between GPC3 or PEG10 long peptide with gp96-NMC/AMP-PNP-bound closed dimer, gp96-NMC/AMP-PNP-bound “twisted V” dimer, or gp96-NMC/ADP-bound “twisted V” dimer. The right panel of **g** or **h** represents the ZDOCK score analysis of the top 10 complex conformations. Ns, not significant; *P < 0.05, **P < 0.01, ***P < 0.001, **** P < 0.0001

We further modeled the structures of the two defined epitopes from GPC3 and PEG10 bound to HLA-A2 using molecular docking analysis. The results showed that both epitopes bind to the active site of the α-subunit of HLA-A2 based on the visible hydrogen bonds and strong electrostatic interactions. Epitope GPC3_152–160_ (KVFGNFPKL) interacts with E63, T73, Y99, Q155, Y159, T163, and W167 via hydrogen bonding in the active cavity (Fig. 3a). Epitope PEG10_229–237_ (ALIGQCIHI) interacts with D77, T80, Y84, R97, W147, and Y159 via hydrogen bonding in the cavity (Fig. 3b). The two epitopes showed low binding energies of −8.0 and −8.8 kcal/mol with HLA-A2, indicating highly stable binding.

We also performed docking modeling of the gp96-dimer with the GPC3 long peptide containing epitope GPC3_152–160_ and PEG10 long peptide containing epitope PEG10_229–237_ using ZDOCK. As shown in Fig. 3c and d, the red region represents a lumen formed by two loops of the *M*-domain (residues 394–407) and a client protein-binding domain (residues 652–678) of the gp96 dimer. The bound polypeptides adopted α-helices and/or β-turns as motifs for gp96 dimer recognition. GPC3 and PEG10 polypeptides bind near the lumen in different manners. The GPC3 long peptide was inserted into the lumen in an α-helical form (Fig. 3c), whereas the PEG10 long peptide bound to the pre-lumenal client protein-binding domain (Fig. 3d). As shown in Fig. 3e and f and Supplemental Table 1, the GPC3 long peptide can form hydrogen bond interactions with the R395, Y678, and D672 residues of gp96, and the PEG10 long peptide can form hydrogen bond interactions with the D672, T675, and S674 residues to achieve stable binding. The binding, hydrolysis, and release of ATP can lead to transitions between different conformations of the gp96-dimer (supplementary Fig. S3). Conformational changes in the polypeptide-binding cavity formed by the two loops of the *M* domain and client protein-binding domain, binding state, and binding energy of the gp96-dimer with GPC3 or PEG10 long peptide may also vary (Fig. 3g and h). Therefore, the process of ATP hydrolysis by the gp96-dimer may affect the binding and release of peptides through conformational changes.

### GPC3 and PEG10 epitopes within Pgp96-bound peptides induce anti-HCC immune response

**Fig. 4:**
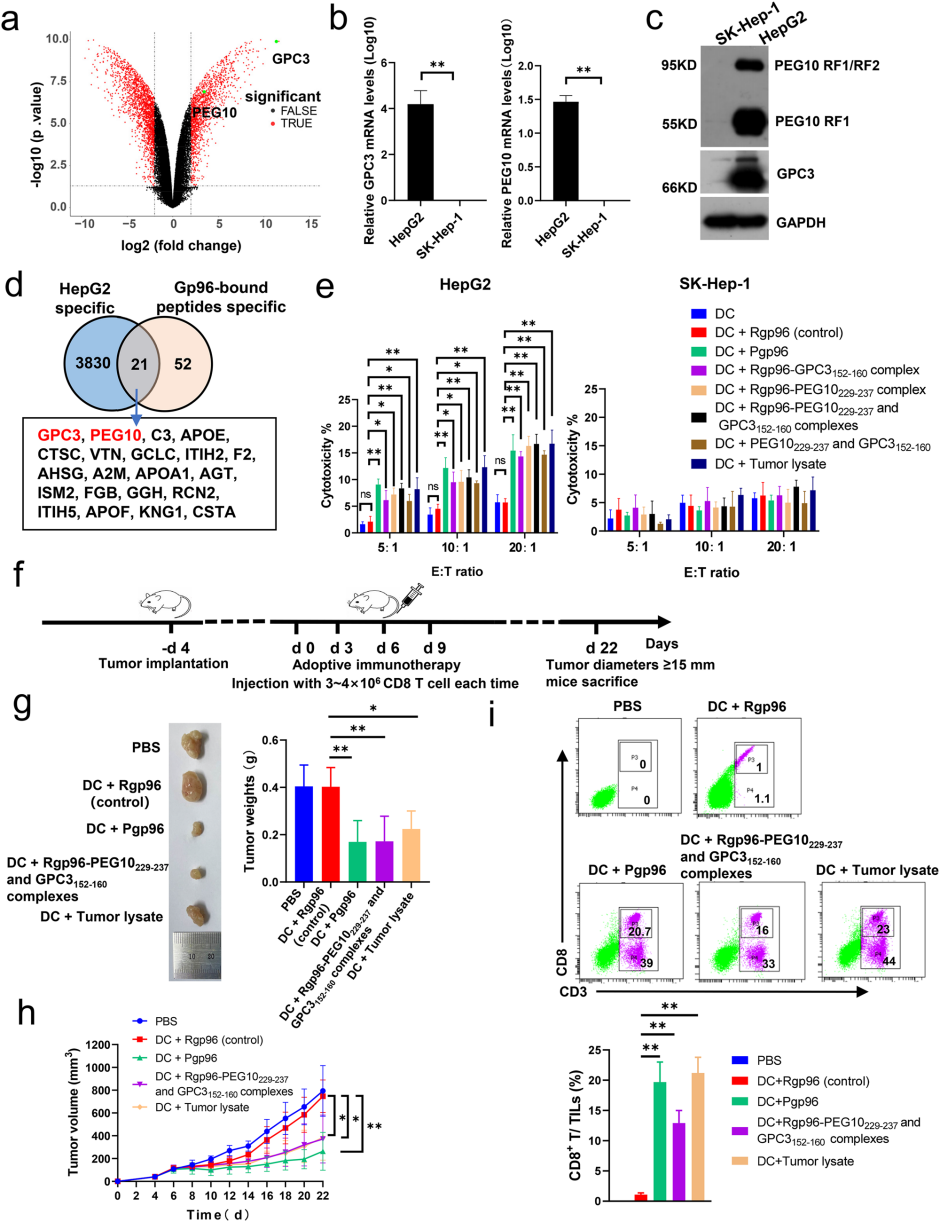
GPC3_152–160_ or PEG10_229–237_-specific *T* cells possess anti-hepatocellular carcinoma (HCC) activity. **a** Volcano plots of differentially expressed genes (DEGs) from HepG2 cells compared to in SK-Hep-1 cells. DEGs were selected by *P* < 0.05 and |log 2 (fold-change)|> 2. The *x*-axis shows the fold-change in gene expression between HepG2 cells and SK-Hep-1 cells, and the *y*-axis shows the significance of the differences. Colors represent different genes: black indicates genes without significantly different expression, and red indicates significantly differentially expressed genes. **b** Relative expression of GPC3 or PEG10 was analyzed using quantitative real-time PCR. Data are the mean ± SD of three replicates. **c** Western blot analysis of GPC3 and PEG10 expression. **d** Venn diagrams illustrating the number of common genes between placental gp96-binding protein genes and genes highly expressed in HepG2 compared to in SK-Hep-1 cells. **e** Female HLA-A2.1/Kb transgenic mice were subcutaneously immunized with BMDCs pulsed with Rgp96, Pgp96, Rgp96-GPC3_152–160_, Rgp96-PEG10_229–237_, Rgp96-GPC3_152–160_ and PEG10_229–237_ complexes, GPC3_152–160_ and PEG10_229–237_ epitopes, or HepG2 tumor lysate, or unpulsed control DCs at weeks 1 and 2. Five days after immunization, splenocytes were harvested for cytotoxic activity with CFSE-labeled HepG2 or SK-Hep-1 cells as target cells. **f** Schedule of animal experiments for *T* cell transfer strategies in HepG2-bearing mice. HepG2 tumor-bearing mice were intravenously injected with GPC3 and PEG10 epitope-, placental gp96-, tumor lysate (positive control)-, or recombinant gp96 (negative control)-induced *T* cells (3–4 × 10^6^/mice) at the indicated times. Mice transferred with PBS were used as a negative control. **g** Representative images and weights of tumors isolated from HepG2-bearing mice. **h** Size of HepG2 tumors was measured at 2-day intervals. **i** The percentage of CD8^+^ *T* cell in tumor-infiltrating lymphocytes was analyzed by flow cytometry. *n* = 5 mice/group. PC, positive control. Ns, not significant; *P < 0.05, **P < 0.01

Using the cancer cell line encyclopedia (CCLE) database, we found that the expression levels of GPC3 and PEG10 were high in HepG2 cells and low in SK-Hep-1 cells (supplementary Fig. S4). Transcriptome analysis, quantitative reverse transcription-polymerase chain reaction (PCR) and Western blot assay showed that the expression levels of GPC3 and PEG10 were much higher in HepG2 cells than in SK-Hep-1 cells (Fig. 4a, b and c). Twenty-one overlapping genes were detected in intersection analysis between protein genes related to placental gp96-bound peptides and protein genes that were highly expressed in HepG2 cells compared to in SK-Hep-1 cells, including GPC3 and PEG10 (Fig. 4d). As shown in Fig. 4e, *T* cells induced by Pgp96, GPC3_152–160_, or PEG10_229–237_ epitope exhibited much higher cytotoxicity against GPC3 and PEG10 high-expressing HepG2 cells than that against GPC3 and PEG10 low-expressing SK-Hep-1 cells. Similar results were observed for HepG2 tumor lysates. In addition, immunization with DC pulsed with sufficient GPC3 and PEG10 epitopes alone could also induce effective antitumor immune response, indicating their potent immunogenicity. We further investigated the ability of the GPC3 and PEG10 epitopes to inhibit HCC in vivo (Fig. 4f). Transfer of epitope-induced CD8^+^ *T* cells resulted in decreased HCC tumor growth and weight in BABL/c-nu mice compared to the effects of control CD8^+^ *T* cells (Fig. 4g and h). Similar tumor inhibitory effects were observed in mice treated with placental gp96- or tumor lysate-induced CD8^+^ *T* cells. We further evaluated CD8^+^ *T* cell infiltration in tumor. As seen in Fig. 4i, compared to control, mice transferred with *T* cells induced by GPC3 and PEG10 epitopes or placental gp96 exhibited much higher percentage of CD8^+^ *T* cells in TILs. This suggests that infiltration of epitopes specific CD8^+^ *T* cells resulted in tumor inhibition.

### Peptides from GPC3 and PEG10 complexed with recombinant gp96 exhibit anti-HCC activity

**Fig. 5:**
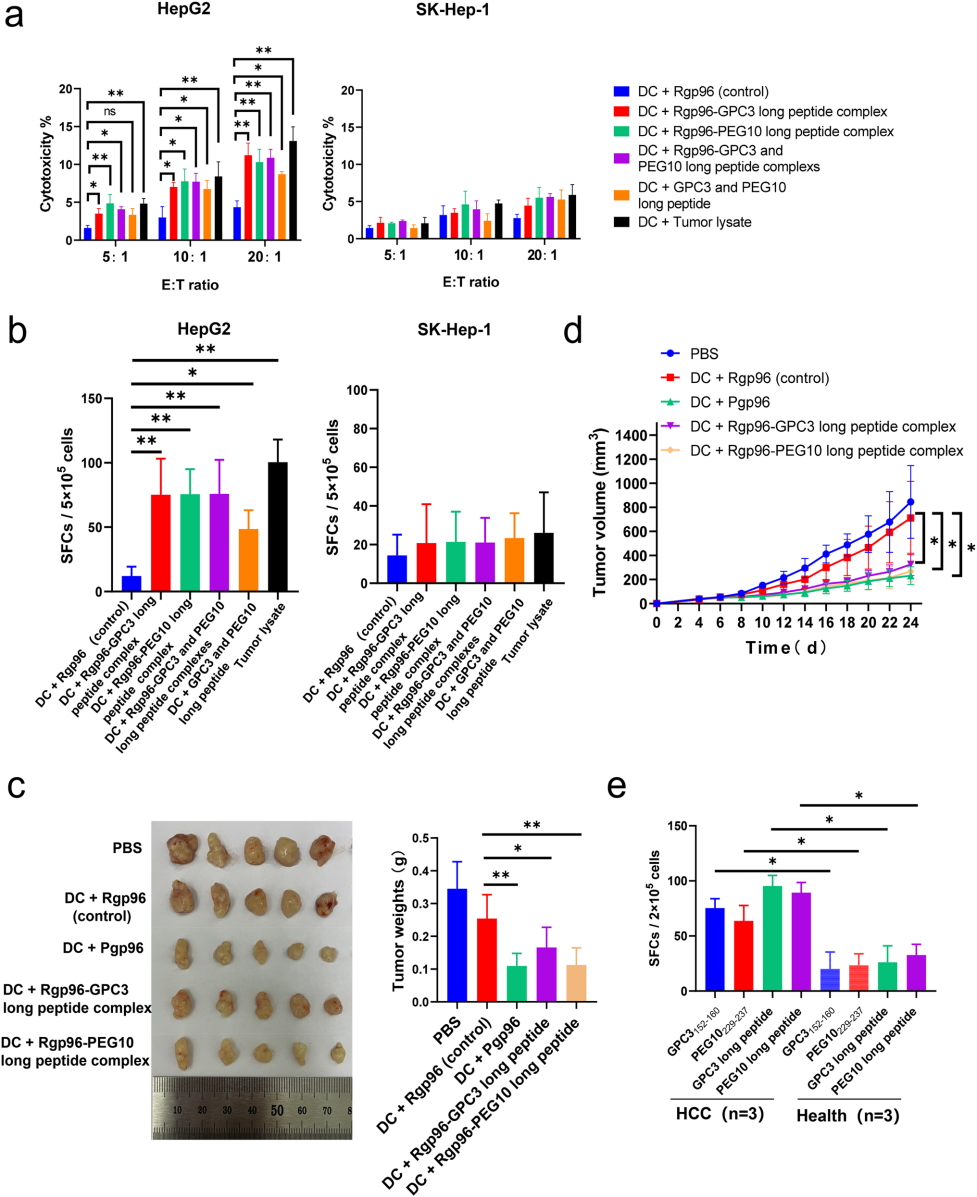
GPC3 and PEG10 peptides associated with placental gp96 have therapeutic effects against hepatocellular carcinoma (HCC). Female HLA-A2.1/Kb transgenic mice were subcutaneously immunized with BMDCs pulsed with Rgp96-GPC3 long peptide (aa 149–185), Rgp96-PEG10 long peptide (aa 201–240) complex, Rgp96-GPC3 and PEG10 long peptide complexes, GPC3 and PEG10 long peptides, or tumor lysate, or Rgp96 alone as control at weeks 1 and 2. At 5 days after the last immunization, splenocytes were harvested and stimulated in vitro for 5 days. **a** Splenocytes were harvested for cytotoxic activity with CFSE-labeled HepG2 or SK-Hep-1 cells as target cells. **b** Splenocytes from immunized mice were stimulated with Hepg2 or SK-Hep-1 whole-cell lysate antigens and assayed in IFN-γ ELISPOT assays. **c**, **d** HepG2 tumor-bearing mice (*n* = 6/group) were intravenously injected with GPC3 or PEG10 long peptide-, placental gp96-, or recombinant gp96 (control)-induced *T* cells (3–4 × 10^6^/mice) at the indicated times. Representative images and weights of tumors from mice with indicated treatment are shown (**c**). Tumor volumes were measured at 2-day intervals (**d**). **e** PBMCs (1 × 10^6^/well) from three HLA-A2 positive HCC patients or healthy individuals (control) were stimulated with the indicated peptides for detection of peptide-specific *T* cells by ELISPOT assay. Results are the mean ± SD. Ns, not significant. Experiments performed twice and showed similar results. *P < 0.05, **P < 0.01

As shown in Fig. 5a, similar to HepG2 tumor lysates, these two GPC3 and PEG10 precursor peptides complexed with recombinant gp96 exhibited specific cytotoxicity for HepG2 cells but not for SK-Hep-1 cells. The ELISPOT assay revealed that GPC3 or PEG10 long peptide-pulsed BMDC immunization resulted in an approximately six or seven fold increase in GPC3 and PEG10 high-expressing HepG2 cells-specific *T* cells compared to control (both *P* < 0.01). In contrast, no obvious specific *T* cell response to GPC3 and PEG10 low-expressing SK-Hep-1 cells-specific *T* cells was detected, indicating that the peptide-induced anti-HCC response was GPC3- and PEG10-specific (Fig. 5b). Immunization with DC pulsed with sufficient GPC3 and PEG10 long peptides alone could also stimulate tumor-specific *T* cell response, indicating their high immunogenicity.

We further assessed the in vivo therapeutic efficacy of GPC3 and PEG10 long peptides in HepG2-bearing mice. As shown in Fig. 5c, d, treatment with CD8^+^ *T* cells induced by GPC3 or PEG10 long peptide showed a significant therapeutic effect against HCC compared to that in the control (all *P* < 0.05 or 0.01).

To address the clinical relevance of the identified GPC3 and PEG10 epitopes and long peptides, we examined peptide-specific CTLs by ELISPOT assay in HCC patients. As shown in Fig. 5e, significantly higher epitope or long peptide-specific CTLs were detected in HLA-A2 positive HCC patients relative to healthy individuals, indicating that these GPC3 and PEG10 epitopes were naturally produced in HCC.

## Discussion

**Fig. 6:**
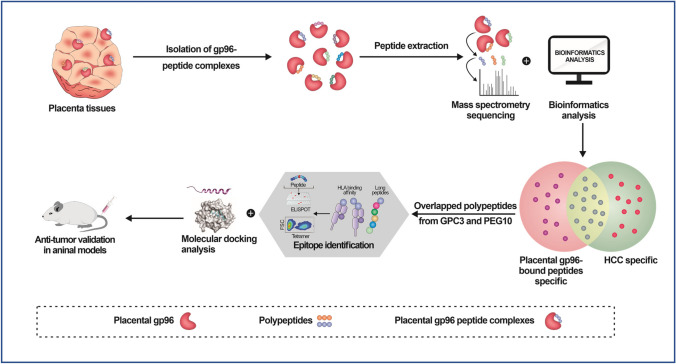
Schematic illustration of using chaperon gp96 to capture and identify antigenic peptides from placenta using mass spectrometry followed by immunopeptidome analysis. Placenta-derived gp96 binds abundant tumor-associated antigen peptides associated with multiple cancers. Antigenic peptides derived from GPC3 and PEG10 were identified from among gp96-bound peptides, which elicit anti-HCC activity

In the current study, using MS-based techniques, we found that abundant tumor-associated antigens that were preferentially expressed in multiple tumor tissues bound to placental gp96, and a large portion of these antigens were primarily associated with HCC. We further established an MS-based immunopeptidomic approach for identifying tumor-specific antigens from the placenta and identified GPC3 and PEG10 as HCC antigens with high immunogenicity recognized by *T* cells (Fig. 6). These results provide insight into the mechanism of the antitumor response mediated by embryonic antigens from fetal tissues or stem cells.

The structure of gp96 contains four domains: *N*-terminal, charged linker, middle, and *C*-terminal. Numerous studies showed that exogenous gp96 mediates the uptake and presentation of its chaperone-like peptides by antigen-presenting cells to MHC-I molecules and activates specific CTLs in vivo [14, 19, 31]. However, the structural interaction between gp96 and the associated peptides remains unclear. A recent study of the gp96: polypeptide interaction revealed that polypeptides associate with the luminal channel that is formed between two loops in the middle domain and client-binding domain of the gp96 dimer [32]. In the present study, we showed that placental gp96 associates with epitopes-harboring long peptides derived from GPC3 and PEG10. The GPC3 and PEG10 long peptides bind to the luminal channel and client-bound domain. Moreover, intramolecular conformational changes induced by ATP hydrolysis may regulate the association and release of polypeptides from gp96 (Fig. 3). Thus, we further uncovered the structural basis of gp96 binding of cellular antigenic peptides and validated that the immunopeptidomic analysis of gp96-bound peptides are an effective approach for antigen capture and identification.

An association between placenta and tumor antigens has been widely reported [33, 34]. In this study, we found that placental gp96 was associated with epitopes-harboring long peptides from GPC3 and PEG10 that elicited anti-HCC *T* cell responses. We further analyzed the source of these antigens in the placenta and found that the GPC3 and PEG10 genes were significantly highly expressed in villous cytotrophoblast cells of normally delivered placentas (Fig. 1f). GPC3 is an oncofetal glycoprotein that binds to the cell membrane via a glycosylphosphatidylinositol (GPI) anchor. GPC3 is expressed in the placenta, numerous embryonic tissues, and various tumors such as HCC but not in the healthy adult liver [35], ovarian carcinoma, and melanomas [36, 37]. GPC3 promotes the growth of HCC cells in a process involving Wnt/β-catenin signaling and is emerging as a potential therapeutic target [38]. PEG10, a paternally expressed imprinted gene that encodes a cytosolic protein, is primarily expressed in the placenta, and its expression levels are elevated in a variety of cancers, including in HCC [39]. PEG10 enhances the proliferation and invasion of cancer cells. In HCC, PEG10 overexpression can improve cell invasion ability and decrease cell apoptosis mediated by SIAH1 [40]. Our current data, along with the above previous studies, clearly demonstrate the similarity of antigen expression patterns between the placenta and tumors and support the design of a new generation of proto-oncogene antigen-based cancer vaccines. Further studies are needed to define new placental antigens and determine their potential functions against cancers, including pancreatic cancer, breast carcinoma, and lung cancer.

In this study, approximately similar specific *T* cell activation and tumor inhibitory effects were observed among mice immunized with PEG10 and GPC3 epitope or long peptide, placental gp96, and tumor lysate (see Figs. 4e–h, and 5a–d). These results suggest that PEG10 and GPC3 specific *T* cells may play a major role in tumor lysate or placental gp96 mediated antitumor response and that placental gp96 shows no obvious superiority over tumor lysate for tumor inhibition. Nevertheless, placenta-derived gp96 that can be readily obtained in high amount for vaccination could allow to provide alternative approach for design of DC tumor vaccines, especially in case of unresectable tumors.

## Conclusions

In summary, we showed that using chaperon gp96 to capture antigenic peptides is an efficient approach for identifying tumor rejection antigens generated in the placenta. GPC3 and PEG10 antigen-pulsed DCs exhibit therapeutic effects against HCC. Large-scale, systematic studies of inherent oncofetal antigens from the human placenta may help reveal novel proto-oncogenes with therapeutic potential against cancer.

### Supplementary Information

Below is the link to the electronic supplementary material.

Supplementary file1 (DOCX 2607 KB)

## Data Availability

All data relevant to the study are available upon reasonable request.
