# Supplementary material for: GPC3 and PEG10 peptides associated with placental gp96 elicit specific T cell immunity against hepatocellular carcinoma
Source: Cancer Immunol Immunother. 2023 Nov 6;72(12):4337–54. doi: 10.1007/s00262-023-03569-2 (PMC10700408; doi:10.1007/s00262-023-03569-2)
Supplement: Supplementary file 1 — Supplementary file1 (DOCX 2607 KB) [file 262_2023_3569_MOESM1_ESM.docx]

**GPC3 and PEG10 polypeptides associated with placental gp96 elicit specific T cell immunity against hepatocellular carcinoma**

Lijuan Qin^1,2,#^, Jiuru Wang^1,2,#^, Fang Cheng^1,2,#^, Jiamin Cheng^4^, Han Zhang^1,2^, Huaguo Zheng^1,2^, Yongai Liu^1,2^, Zhentao Liang^1,2^, Baifeng Wang^1,2^,Changfei Li^1^, Haoyu Wang^1,2^, Ying Ju^1,^*, Huaqin Tian^3,^*, Songdong Meng^1,2,^*

**Supplementary Information**


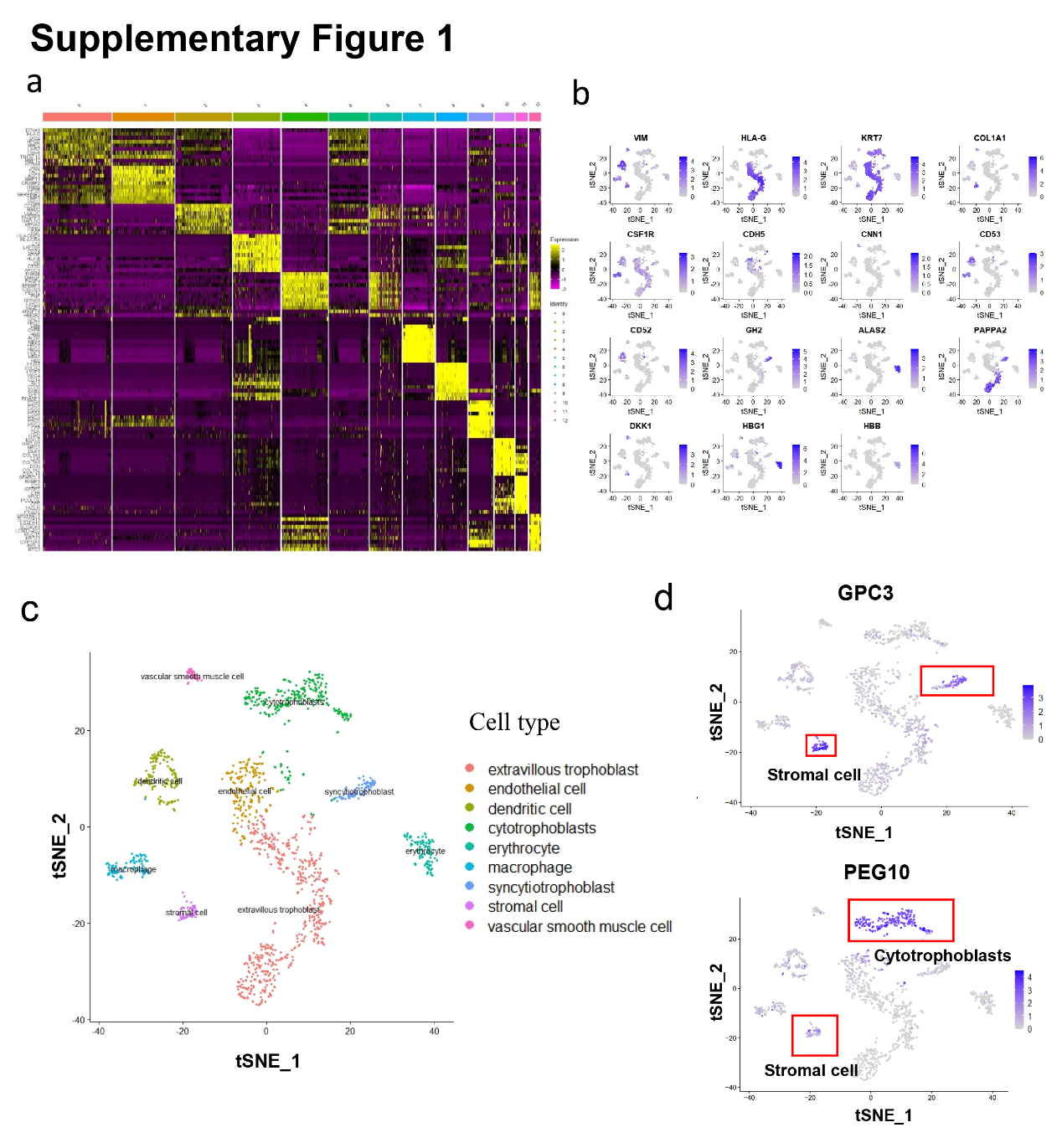


**Supplementary Figure S1. Analysis and annotation of single-cell transcriptome data of human early placentas.** Related to Figure 1. (**a**) Heatmap showing the characteristic genes of different cell populations. The t-stochastic neighborhood embedding (t-SNE) algorithm in the R package Seurat was used to group cells and draw heat maps of cell specific genes of different populations to test the reliability of grouping. (**b**) Expression of different marker genes. (**c**) Cell population mapping and annotation after 1567 single-cell t-SNE dimensionality reduction. (**d**) GPC3 and PEG10 gene expression in different cell populations in single-cell transcriptome data. A darker color indicates a higher expression level in the cells. The cell population circled by the red frame expressed high levels of GPC3 and PEG10.


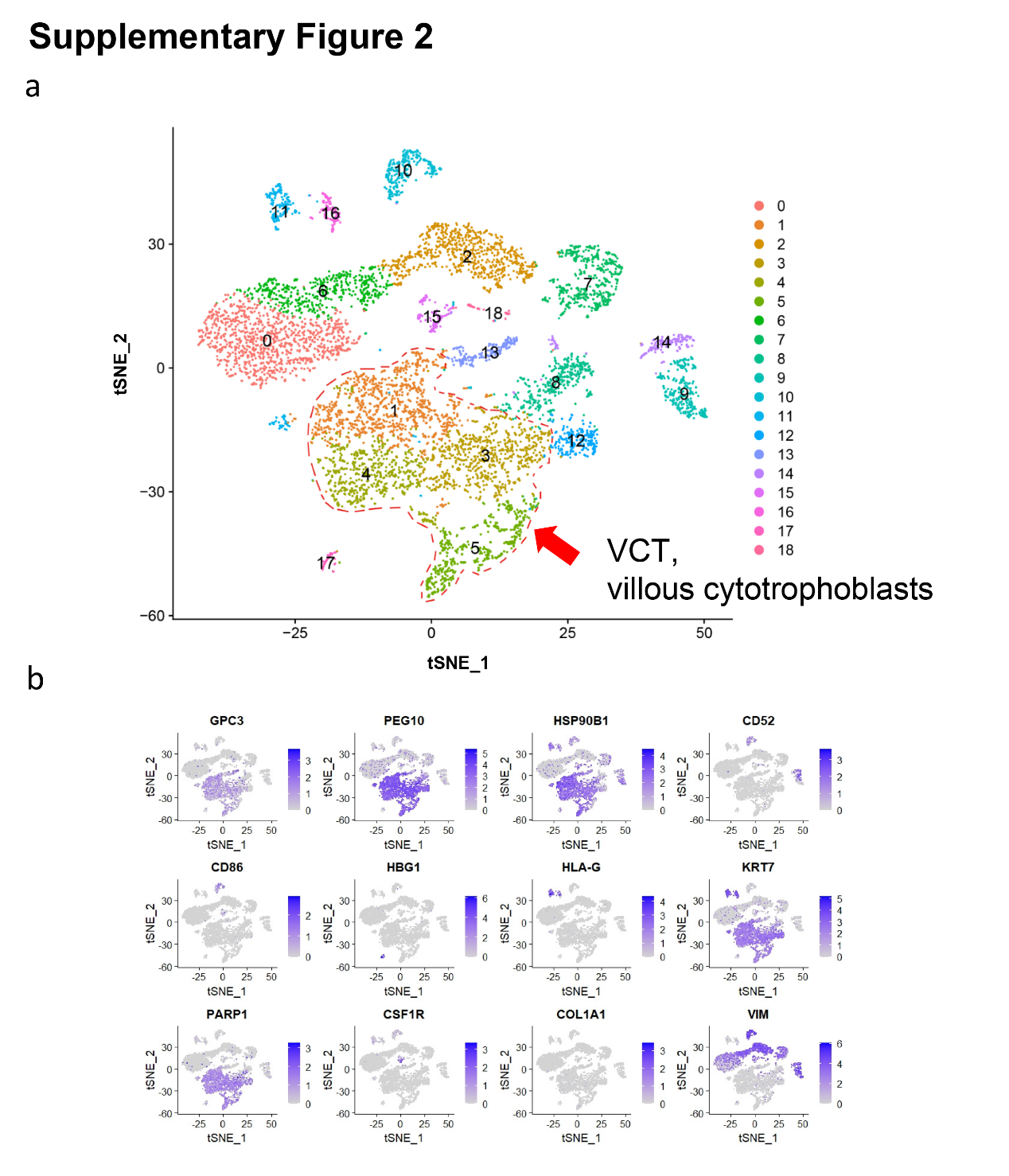


**Supplementary Figure S2. t-Stochastic neighborhood embedding** **(t-SNE) dimensionality reduction and gene expression of single-cell sequencing data of normal delivery placenta.** Related to Figure 1. (**a**) Expression map of different genes visualized using t-SNE dimensionality reduction; red dashed cell group is villous cytotrophoblast cells. (**b**) Expression of different marker genes was visualized using the FeaturePlot() function in the Seurat package. A darker color indicates a higher expression level in the cells.


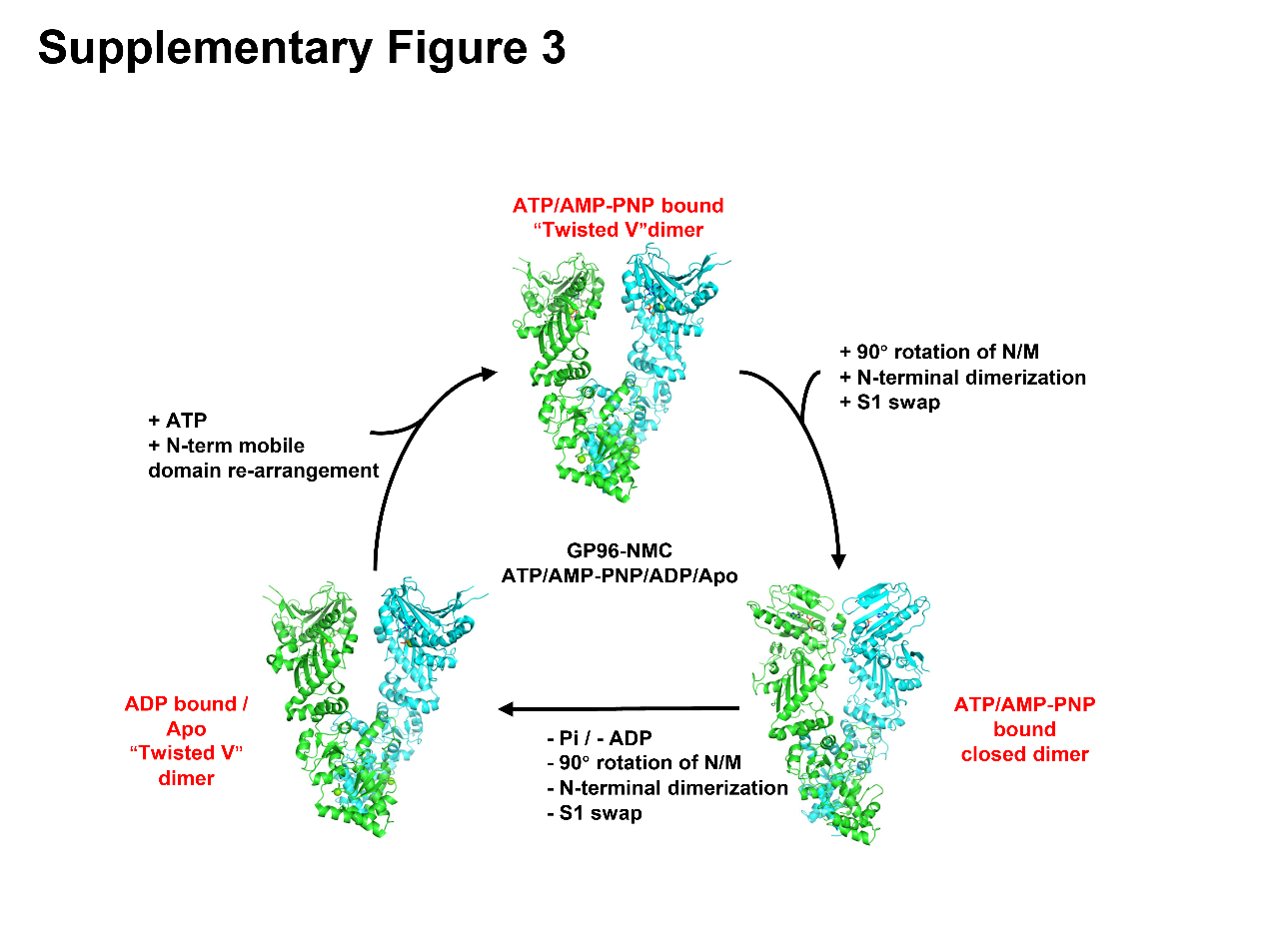


**Supplementary Figure S3. Schematic diagram of the mechanism that ATP hydrolysis of gp96 leads to conformational change.** Related to Figure 3. In the state of the "twisted V" dimer, gp96's N-terminal domain can bind ATP/AMP-PNP (non-hydrolytic), leading to a conformational change in the N-terminal domain and 90° rotation around the N-terminal/middle domain interface, thus promoting N-terminal dimerization. At this time, a closed dimer binding ATP/AMP-PNP is formed to prepare for ATP hydrolysis. After ATP hydrolysis and ADP release, the transient N-terminal dimer decomposes and re-rotates around the N-terminal/middle domain interface to form a mobile N-terminal domain.


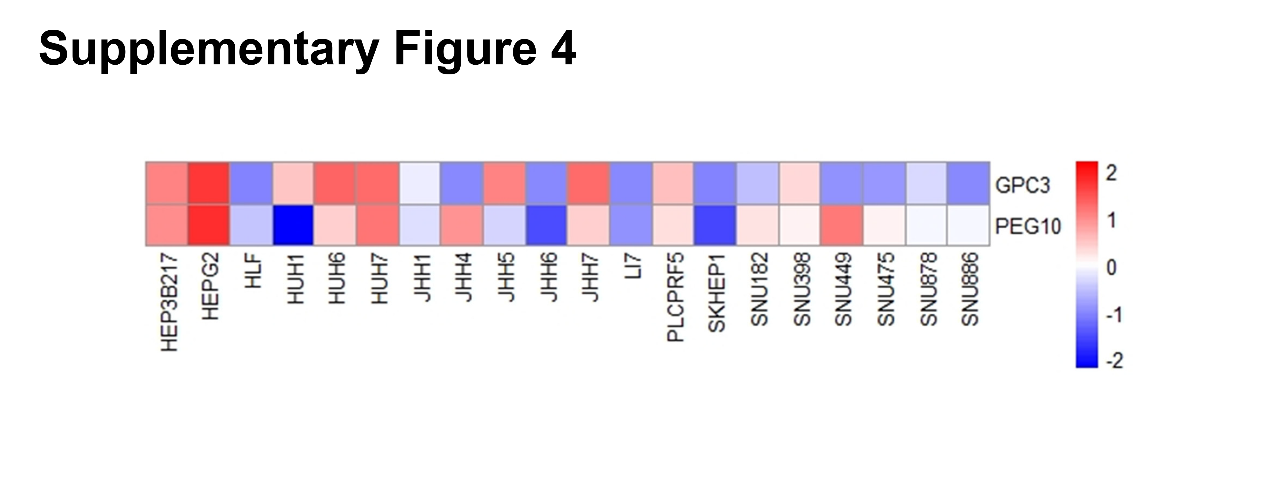


**Supplementary Figure S4. Heat map of expression levels of GPC3 and PEG10.** Related to Figure 4. The expression profile data of different hepatocellular carcinoma cell lines were downloaded from the Cancer Cell Line Encyclopedia cancer database and analyzed.

**Supplementary Table S1. Results of molecular docking ZDOCK, discovery studio 2019**

| **Receptor** | **Ligand** | **ZDOCK score** | **Hydrogen bond interaction** | **Electrostatic interaction** | **Hydrophobic** |
| --- | --- | --- | --- | --- | --- |
| gp96-dimer | GPC3_149-185_ | 16.04 | A:ARG395:NH1 - C:GLU238:OE2 | A:ARG395:NH2 - C:GLU238:OE1 | C:VAL207:CG1 - A:TYR652 |
|  |  |  | A:ARG587:NH2 - C:ASP204:OD2 | A:ARG587:NH1 - C:ASP204:OD1 | C:ARG203 - A:PRO581 |
|  |  |  | A:GLN668:NE2 - C:ILE215:O |  | C:VAL219 - A:MET662 |
|  |  |  | A:TYR678:OH - C:PHE208:O |  | A:TYR575 - C:VAL219 |
|  |  |  | C:PRO212:CD - A:TYR678:OH |  | A:TRP654 - C:ILE215 |
| gp96-dimer | PEG10_201-240_ | 19.6 | A:TYR652:OH - C:LEU202:O |  | A:MET662 - C:ILE235 |
|  |  |  | A:THR675:N - C:ILE235:O |  | A:ILE673 - C:ILE237 |
|  |  |  | A:THR675:OG1 - C:ILE235:O |  | A:TYR652 - C:LEU227 |
|  |  |  | A:TYR677:OH - C:GLY209:O |  | A:TRP654 - C:ILE235 |
|  |  |  | A:TYR678:OH - C:ILE231:O |  | A:TYR677 - C:LEU210 |
|  |  |  | C:SER228:OG - A:GLN578:O |  | A:TYR678 - C:CYS234 |
|  |  |  | A:SER674:CB - C:GLN233:O |  |  |

**Supplementary Table S2. Prediction of HLA-DRB1-restricted epitopes of placental gp96-bound peptides**

| **Gene name** | **Peptide sequences (Position)** | **HLA type** | **Epitopes** | **Position** | **Score** | **IEDB  percentile  rank** |
| --- | --- | --- | --- | --- | --- | --- |
| PEG10 | AHLATYTEFVPQIPGYQTYPTYAAYPTYPVGFA (620-652) | HLA-DRB1*15:01 | PTYAAYPTYPVG | 639-650 | 0.7474 | 0.04 |
|  |  |  | PGYQTYPTYAAY | 633-644 | 0.5497 | 0.16 |
|  |  | HLA-DRB1*08:03 | PTYAAYPTYPVG | 639-650 | 0.5331 | 0.22 |
|  |  |  | PGYQTYPTYAAY | 633-644 | 0.3763 | 0.65 |
|  |  | HLA-DRB1*09:01 | PTYAAYPTYPVG | 639-650 | 0.4941 | 0.27 |
|  |  | HLA-DRB1*07:01 | PTYAAYPTYPVG | 639-650 | 0.4304 | 0.5 |
|  | ALIDQYHEGLSDHIQEELSHLEVAKSLSALIGQCIHIERR ^d^ (201-240) | HLA-DRB1*07:01 | HLEVAKSLSALIGQ | 220-33 | 0.6734 | 0.87 |
|  | DHRLVDPHIEMIPGAHSIPSGHVYSLSEPEMAALR (524-558) | HLA-DRB1*09:01 | GHVYSLSEPEMAA | 544-556 | 0.581 | 0.95 |
| GPC3 | RDLKVFGNFPKLIMTQVSKSLQVTRIFLQALNLGIEV ^d^ (149-185) | HLA-DRB1*15:01 | RDLKVFGNFPKL | 149-160 | 0.3206 | 0.37 |
|  | NVLLGLFSTIHDSIQYVQKNAGKLTTTIGKLCAHSQQRQYRSAYYPEDLFIDKKVLKV (264-321) | HLA-DRB1*08:03 | DSIQYVQKNAGK | 275-286 | 0.5635 | 0.16 |
|  |  |  | EDLFIDKKVLKV | 310-321 | 0.485 | 0.31 |
|  |  |  | SIQYVQKNAGKL | 276-287 | 0.4731 | 0.35 |
